# Novel *BRAF* fusion in Erdheim–Chester disease with pulmonary manifestations: Importance of RNA‐based testing and response to MEK inhibition

**DOI:** 10.1111/his.70170

**Published:** 2026-05-04

**Authors:** Igor Odintsov, Henry D. Tazelaar, Jerry C. Lee, Lloyd E. Damon, Walter Patrick Devine, Kirk D. Jones

**Affiliations:** ^1^ Department of Pathology University of California San Francisco San Francisco California USA; ^2^ Department of Anatomic Pathology Mayo Clinic Arizona Scottsdale Arizona USA; ^3^ Helen Diller Family Comprehensive Cancer Center University of California San Francisco San Francisco California USA

**Abbreviations**
CHIP: clonal haematopoiesis of indeterminate potential
ECD: Erdheim–Chester disease
ILD: interstitial lung disease
MAPK: mitogen activated protein kinase
NGS: next‐generation sequencing

## Introduction

Erdheim–Chester disease (ECD) is a rare form of non‐Langerhans cell histiocytosis, which occurs predominantly in the adult population.^1^ The disease most commonly affects bone, but can present itself in other sites, including CNS, orbit, skin, heart and lung.^1^ ECD frequently manifests as progressive interstitial lung disease (ILD).^2^ Due to its rarity and ILD‐like clinicoradiological presentation, diagnosis of ECD can be challenging on a small lung biopsy.

ECD is a clonal process driven by activating somatic genomic alterations in the mitogen activated protein kinase (MAPK) pathway, most commonly *BRAF* p.V600E, followed by mutations in *MAP2K1*, *MAP2K2*, *KRAS*, *NRAS*, *ARAF* and others.^3^ Genomic testing can provide additional information, supporting the diagnosis in uncertain cases. Moreover, many of the MAPK pathway alterations are targetable with FDA‐approved systemic therapies (e.g. *BRAF* p.V600E responds to BRAF kinase inhibitors such as vemurafenib^4^). Given that untreated ECD can result in end‐stage lung fibrosis necessitating lung transplantation, targeted therapies offer hope to delay or halt disease progression. Routine genomic testing of pulmonary ECD is therefore imperative for widespread adoption of this treatment strategy.

We present a case of ECD diagnosed via transbronchial lung cryobiopsy. We demonstrate pertinent histological features and report a novel *BRAF* fusion not documented in ECD before.

## Case

### Presentation

A male in his 60s presented with persistent constitutional and respiratory symptoms. Diagnostic imaging via chest CT showed diffuse bronchial and septal thickening with subpleural consolidations. Subsequent PET/CT demonstrated multisystemic, FDG‐avid disease involving the lungs, bone and retroperitoneum. Although multifocal hypermetabolic skeletal activity was identified, no characteristic sclerotic bone lesions were present.

### Histopathology

**Figure 1:**
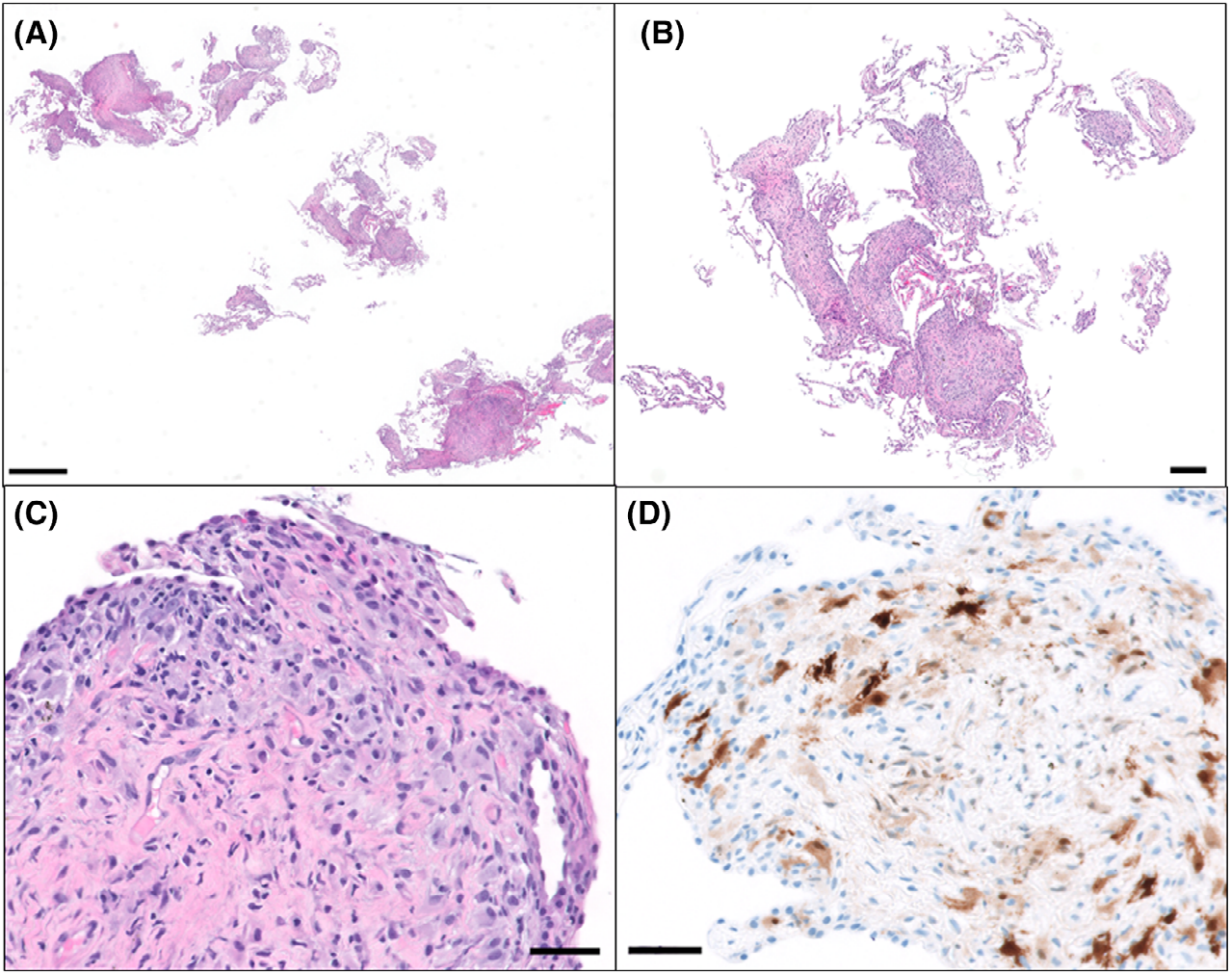
Endobronchial cryobiopsy demonstrating characteristic features of Erdheim–Chester disease. (**A–C**) Haematoxylin and eosin‐stained sections, showing expansion of the interstitium with sparse histiocytic proliferation and admixed inflammatory cells. The histiocytes show pale‐eosinophilic cytoplasm and bland monotonous nuclei. (**D**) Immunoperoxidase staining with S100 antibody highlights the histiocytic proliferation. Size bars: A—1 mm, B—200 μm, C and D—50 μm.

Transbronchial cryobiopsy was performed and demonstrated expansion of bronchovascular bundles and interlobular septa by a proliferation of histiocytes with pale eosinophilic cytoplasm in a fibrotic stroma (Figure 1A–C). Frequent plasma cells and lymphocytes were admixed with the histiocytes. Immunoperoxidase studies demonstrated that the histiocytes are positive for S100 (Figure 1D), CD68 and factor XIIIa and negative for CD1a, AE1/AE3 and CAM5.2. Acid‐fast stain and Grocott's methenamine silver stain demonstrated no infectious microorganisms. Although S100 positivity is uncommon in ECD, the absence of CD1a expression distinguishes this proliferation from Langerhans cell histiocytosis; the fibrotic stroma and absence of emperipolesis are atypical for Rosai–Dorfman disease.

### Genomic Profiling

**Figure 2:**
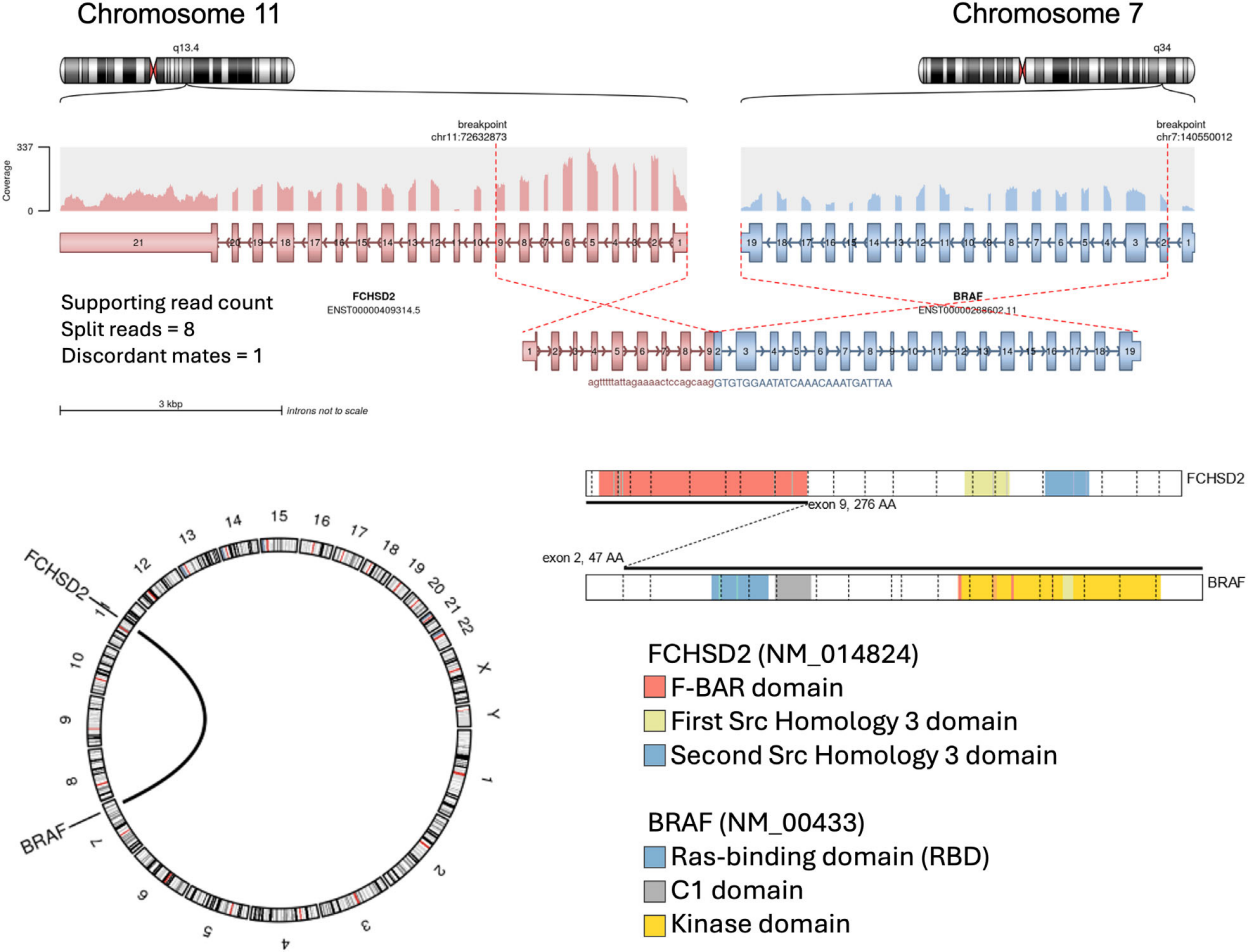
Graphical visualization of the *FCHSD2* exon 9:: *BRAF* exon 2 fusion identified through targeted next‐generation RNA sequencing. Utilized transcripts: FCHSD2 ENST00000409314.5.5; BRAF ENST00000288602.11.

*BRAF* single‐gene mutation assay did not detect a *BRAF* p.V600E mutation. Hybrid‐capture DNA‐based next‐generation sequencing (NGS) tumour panel UCSF500 showed *SRSF2* p.P95L (variant allele frequency [VAF] = 0.16) and *TET2* c.4573 + 1G>A (VAF = 0.28) variants, but no alterations in the MAPK pathway. To further explore a potential MAPK driver, RNA‐based sequencing was performed and demonstrated the presence of a chimeric in‐frame transcript between *FCHSD2* exon 9 and *BRAF* exon 2 (Figure 2).

### Treatment

The patient began a regimen of daily cobimetinib (21 days of each 28‐day cycle) and prednisone 40 mg. Clinical follow‐up showed marked improvement in energy levels and respiratory status. Correspondingly, a PET/CT scan demonstrated the complete or near‐complete resolution of FDG‐avid lesions across the lungs, bone and retroperitoneal lymph nodes.

## Discussion

Erdheim–Chester disease (ECD) is a rare systemic histiocytosis characterized by the accumulation of clonal histiocytes. Pulmonary involvement can mimic interstitial lung disease, often requiring biopsy for diagnosis. Histologically, ECD may be mistaken for non‐specific reactive processes; thus, maintaining a high index of clinical suspicion is critical.

ECD is driven by activating somatic alterations in the MAPK pathway, most commonly the *BRAF* p.V600E mutation. Rarely, BRAF fusions have been reported in this entity.^5^, ^6^ While most *BRAF* fusions involve exons 8–11,^7^ we identified a novel *FCHSD2*::*BRAF* rearrangement involving exon 2. Physiologically, Class II fusions are postulated to result in RAS‐independent dimerization. In this case, the FCHSD2 FCH‐BAR domain likely facilitates oncogenic activation by tethering nearly full‐length BRAF to the plasma membrane and promoting oligomerization.^8^

A critical diagnostic takeaway is the necessity of RNA‐based sequencing. *BRAF* exon 2 fusions involve a large intron 1 (~75,000 bp) that is typically excluded from standard DNA‐based hybridization‐capture panels. Consequently, DNA sequencing failed to detect the fusion in our patient. RNA sequencing is not only more efficient at capturing these breakpoints but also more sensitive in specimens with low tumour content—a common challenge in pulmonary ECD where neoplastic cells are sparsely distributed among inflammatory infiltrates.

Therapeutically, *BRAF* p.V600E mutations respond well to vemurafenib, but *BRAF* fusions generally do not. However, MEK1/2 inhibitors like cobimetinib provide a ‘one‐size‐fits‐all’ approach by targeting the pathway downstream of RAS and RAF. Our patient demonstrated a rapid, multisystemic response to cobimetinib, consistent with clinical trial data showing high response rates in histiocytic neoplasms regardless of the specific driver mutation.

Finally, the detection of *SRSF2* and *TET2* variants in our case likely reflects underlying clonal haematopoiesis of indeterminate potential (CHIP) or an associated myeloid malignancy, both of which occur with high frequency (up to 42.5%) in ECD patients.^9^

In conclusion, BRAF exon 2 fusions are likely underreported due to the limitations of DNA sequencing. Clinicians should utilize RNA‐based assays when DNA‐based panels are negative. This case confirms that *BRAF* fusion‐positive ECD remains highly responsive to MEK inhibition.

## Conflicts of interest

The authors declare no conflicts of interest.

## Data Availability

The data that support the findings of this study are available from the corresponding author upon reasonable request.
